# Multiple Electrophysiological Markers of Visual-Attentional Processing in a Novel Task Directed toward Clinical Use

**DOI:** 10.1155/2012/618654

**Published:** 2012-11-24

**Authors:** Julie Bolduc-Teasdale, Pierre Jolicoeur, Michelle McKerral

**Affiliations:** ^1^Centre for Interdisciplinary Research in Rehabilitation and Lucie-Bruneau Rehabilitation Centre, 2275 Laurier Avenue East, Montreal, QC, Canada H2H 2N8; ^2^Centre for Research in Neuropsychology and Cognition, University of Montreal, C.P. 6128, Succursale Centre-Ville, Montreal, QC, Canada H3C 3J7; ^3^Department of Psychology, University of Montreal, C.P. 6128, Succursale Centre-Ville, Montreal, QC, Montréal, QC, Canada H3C 3J7

## Abstract

Individuals who have sustained a mild brain injury (e.g., mild traumatic brain injury or mild cerebrovascular stroke) are at risk to show persistent cognitive symptoms (attention and memory) after the acute postinjury phase. Although studies have shown that those patients perform normally on neuropsychological tests, cognitive symptoms remain present, and there is a need for more precise diagnostic tools. The aim of this study was to develop precise and sensitive markers for the diagnosis of post brain injury deficits in visual and attentional functions which could be easily translated in a clinical setting. Using electrophysiology, we have developed a task that allows the tracking of the processes involved in the deployment of visual spatial attention from early stages of visual treatment (N1, P1, N2, and P2) to higher levels of cognitive processing (no-go N2, P3a, P3b, N2pc, SPCN). This study presents a description of this protocol and its validation in 19 normal participants. Results indicated the statistically significant presence of all ERPs aimed to be elicited by this novel task. This task could allow clinicians to track the recovery of the mechanisms involved in the deployment of visual-attentional processing, contributing to better diagnosis and treatment management for persons who suffer a brain injury.

## 1. Introduction

Event-related brain potentials (ERPs) permit a relatively inexpensive and precise way to assess the integrity and the efficiency of specific stages or levels of information processing via the amplitude and latency of well-established components of the ERP [1, 2]. With excellent temporal resolution, ERPs allow for the simultaneous measurement of sensory, perceptual, and cognitive processes involved in the performance of simple tasks. By tracking multiple intermediate stages of processing, ERPs possess an advantage over reaction time as a measure of performance. ERPs have the potential to provide an entire set of measures, each associated with different intermediate processes, instead of reflecting the sum of all the stages from sensation, to response generation involved in task performance [2]. 

Experimental paradigms have traditionally been designed to study one or two particular visual or cognitive processes, in association with their event-related components. For example, *oddball *paradigms have been widely used to evoke the P3, a component peaking at around 350 ms post stimulus, arguably reflecting the updating of information in working memory [3]. Paradigms intended to measure earlier-level integrative visual processes, such as texture segregation, have also been developed. Such paradigms have been shown to be sensitive to various clinical diseases such as schizophrenia [4], attention deficit disorders (ADDs) [5], and acquired neurological insults such as mild traumatic brain injury (TBI) [6–10]. 

In the particular case of mild TBI, which mostly involves a diffuse pathophysiological process that cannot be measured with traditional structural brain imaging measures used in the clinic (e.g., CT-scan, MRI), we now know that it can hinder different brain functions, from earlier visual processing to more complex cognitive processing such as attention and working memory. The importance of mild TBI in the clinical context results from the variety and complexity of individual cases and from a very high incidence, estimated at 600 cases per 100 000 population [11]. Similarly, other mild brain injuries (e.g., mild cerebrovascular stroke) also have a high incidence (almost as prevalent as mild TBI in the case of mild stroke) and can lead to persistent functional problems related to sensory, motor, and cognitive information processing [12]. As such, there is a need for a sensitive and objective tool to obtain precise and rapid measures of the underlying functional impairments. 

Although ERPs have many advantages, an important weakness of typical cognitive electrophysiological paradigms is the long duration of tests needed to provide reliable estimates of component characteristics. A typical task requires a large number of trials, and evaluating multiple functions would require several tasks, which collectively would require several hours of testing. This approach is not realistic for routine assessments in a hospital or medical setting. The goal of this study was thus, to develop a new approach allowing us to evaluate several aspects of visual information processing and attention in a single-test session. 

Here we explored an approach based on measures derived from the electroencephalogram (EEG) in order to capitalize on the distinct advantages of ERPs in bridging function-brain barrier. We created a new task that measures the different steps involved in the deployement of visuospatial attention that are at risk to be impaired after a mild TBI. The task allows us to measure the integrity of the interaction between these processes in the deployment of visuospatial selective attention, from the earlier visual evoked potentials (P1, N1, and P2) to the visuocognitive event related potentials (N2pc, SPCN, P3a, and P3b). 

The P1 is a positive component that usually culminates between 80 and 120 ms after stimulus which is thought to originate from the fusiform gyrus [13]. This component might reflect a facilitation effect for stimuli presented at an attended localisation [14]. Another early component is the visual N1, a negative component peaking at 150–200 ms after stimulus, which has distributed sources in lateral extrastriate cortex [15]. Although the N1 is strongly affected by stimulus characteristics (e.g., luminance) and spatial attention [16], suggesting strong bottom-up influences, it can also reflect later processes, such as discrimination processes within the focus of visual attention [17] or central attention [18]. 

A component of the ERP, the N2pc, can be used as an index of the deployment of visual spatial attention. The N2pc is a negative ERP component culminating around 200–250 ms that reflects, where spatial attention is focused in that it is more negative at electrode sites that are over the posterior contralateral hemisphere relative to the visual hemifield towards which attention is deployed [19]. This component appears to reflect an important attentional filtering process, that likely facilitates further processing of selected visual information [19–21]. Stimuli selected for further processing usually pass into visual working memory and elicit a later component called the sustained posterior contralateral negativity (SPCN). The SPCN usually begins shortly after the N2pc, at about 300 ms post stimulus, and has an amplitude that increases with the number of representations held in visual working memory [20, 22]. The sources of the SPCN are in lateral occipital and superior parietal cortex [23, 24].

Another important aspect of selective attention is the presence of a mechanism that controls the orientation of attention toward the source of a visual signal [25, 26]. This orienting process implies disengagement, movement, and reengagement processes each interrelated and associated with arguably distinct neural networks [27]. One ERP component that is thought to reflect the ability to disengage attention in order to reorient the resources toward a novel stimulus is the P3a [28, 29]. This component is classically evoked by a three-stimulus* oddball* paradigm in which one type of stimuli is a rare, task-irrelevant, nontarget stimulus [29–36]. The amplitude of this component is greater at frontal and central electrodes. The generators for this component have been argued to be in the prefrontal and frontal areas [29, 30]. The supramarginal gyrus and the hippocampal formation also play a role in the generation of this component [29, 30]. 

Classic oddball paradigms also typically elicit greater P2 and P3b components in response to the rare stimulus. This experimental paradigm involves the discrimination between frequent (standards) and rare stimuli (targets) [37]. The P3b appears to reflect mechanisms that keep track of task-relevant stimuli, particularly when the contents of working memory need to be updated [29, 30, 34, 37]. The integrity of these mechanisms is reflected by the amplitude and latency of the P3b [36]. The latency of the P3b wave can be taken as an indication of the duration of the processes that took place before and up to the discrimination required to determine which stimulus is frequent and which is rare, given that, stimulus frequency has a large effect on the P3b [38]. This component is thus, of major interest as an index of the integrity of stimulus encoding and classification processes, likely reflecting in part neural mechanisms in the supramarginal gyrus of the temporo-parietal junction and possibly the hippocampic formation [29, 30]. 

In the presence of a context in which making a response is the prepotent behavior, stimuli for which one must omit a response are typically accompanied by a relatively large fronto-central negativity, in the N2 time range. This component is often called the no-go N2, or the control-related N2 [39]. This component is widely argued to reflect the activation of the anterior cingulated cortex, in various paradigms (e.g., [40]). 

By tracking several different steps of visuospatial attention in the context of a single task, this new paradigm allows for a relatively rapid measure of the functional integrity of numerous perceptual, memory, and executive control functions in a test that would be sufficiently short for routine use in the clinic. 

## 2. Methodology 

### 2.1. Participants

Twenty-four (24) normal participants were tested for this study. From that number, 19 participants were kept for analysis (10 males, mean age 25.9 yrs, SD 6.61). Five participants were removed from the analysis because of the presence of artifacts (mainly because of loss of trials (more than 50% of trials) due to eye blinks; 2 were rejected because of very large alpha oscillations). Participants were recruited through publicity posted in a community center. Exclusion criteria consisted of any clinical neurological (history of brain trauma, seizures, attention deficit disorder, or learning disability) or psychiatric history (depression, anxiety disorder, or other). Participants also had to have normal or corrected-to-normal vision. The project was approved by the research ethics committee of the Center for Interdisciplinary Research in Rehabilitation of Greater Montreal, and the subjects gave their written informed consent prior to testing. Subjects have also received a small financial compensation for their participation. 

### 2.2. Procedure

To evoke all the components of interest, participants went through five blocks of 80 experimental trials (400 trials total), after a practice block of 40 trials. Participants were 1.14 meters from a computer screen used to present visual stimuli and were instructed to maintain fixation on a point at the centre of the screen and to avoid eye movements or blinks during trials. The presentation of the stimuli was initiated when the participant pressed the spacebar of a computer keyboard with both thumbs, in order to avoid possible motor lateralization artifacts. There was a 600 ms mean time interval (400 ms to 800 ms, 600 ± 200 ms, random jitter) between the pressing of the spacebar and the occurrence of the stimulus. The target stimulus was then presented for 150 ms. Participants had 2000 ms to respond. Pauses could be taken at any time during trial blocks and were encouraged between blocks but left at the discretion of the participant. 

**Figure 1:**
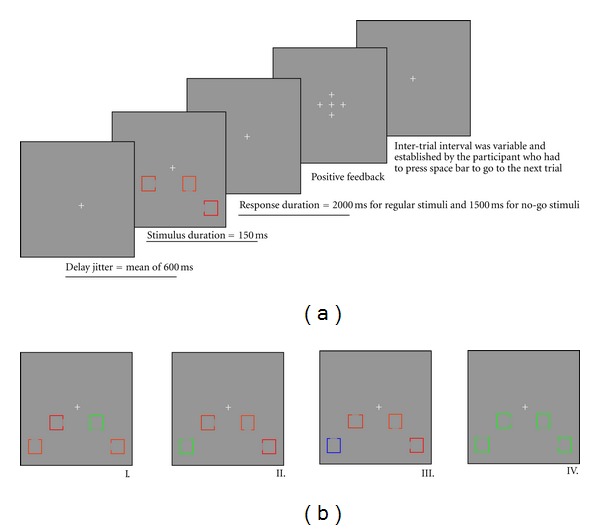
Experimental paradigm. Image (a) presents the experimental design. Image (b) presents the different type of stimuli: I. frequent position of the square opening (standard stimulus), II. infrequent position of the square opening (target stimulus), III. infrequent colour of the target stimulus, and IV. same colour stimulus. Colour of different type of stimuli was counterbalanced among subjects.

The main task was to indicate the location of a small gap in the side of a coloured square, as illustrated in Figure 1. On target-present trials, the target square was the odd-coloured square in a set of four (see [6, 18] for previous work with similar paradigms). On target-absent trials, all the squares had the same colour, and this colour was not used in target-present trials; furthermore, these trials occurred only on 10% of trials. As can be seen in Figure 1, the target square, when presented, was either in the lower left or lower right visual field, in order to maximize the amplitude of the N2pc (see [41]). This manipulation, which consisted in controlling the appearance of the target either to the left or to the right of the fixation point, also allowed us to evoke the SPCN, which is also lateralized, and provides a measure of the integrity of encoding of the target in visual short-term memory. 

### 2.3. Stimuli

Each of the four squares subtended a visual angle of 1° × 1° degree with an opening of 0.33° degree on one side. Two squares were presented on each side of the fixation point. The center of the squares nearest to the fixation point was 1.5° below and 3.5° to the left or the right of the fixation point. The center of the far squares was 3° below and 5° to the left or right of the fixation point. The target square was presented equally often at each of the four possible positions (near-left of fixation, near-right of fixation, far-left of fixation, and far-right of fixation). The squares were one of three different colours; blue, red, or green. The ERPs were evoked by manipulating the position of the opening of target squares, their colour, and the frequency of their occurrence. All these parameters were counterbalanced amongst participants such that the specific colours were not confounded with the various conditions in the experiment (the colours illustrated in Figure 1 represent the colour assignments for one of the many counterbalancing groups). The intensity of the different colours of squares was calibrated to be equiluminant with a chroma meter (Minolta CS100) in order to control for low-level sensory responses. 

Although the position of the gap in the target square was equally often in each of the four possible positions (4 sides of the square), the instructions mapped these positions to one of two responses, one of which had a probability of 25% and one a probability of 75% (e.g., some participants had to decide whether the gap was at the top of the square [25%] or not at the top [75%]). This created a relative frequency difference across the two responses, despite the fact that each gap location was equally probable. The expectation was that this relative frequency manipulation, inspired by classical *oddball* paradigms [3], would elicit a larger P3b for the less frequent response relative to the P3b in the more frequent response condition [37]. Participants had to detect the position of the opening and then press the “m” key, if the position was, say, upwards, or the “c” key, if the opening was on any of the other three sides of the square. Thus, all possible answers required the same action: pressing a key. To avoid motor lateralization effects, the response hand associated with the detection of the target was counterbalanced between participants. 

To evoke the P3a, an attempt was made according to the guidelines set by the literature on the classical *oddball *paradigm. In these studies, the P3a is evoked using irrelevant stimuli; generally squares presented among frequent large (standard) and small rare (target) circles. In order to respect this principle, we created a condition in which the target stimulus was presented in a rare and in a totally new colour, in 25% of the go-trials. Expect for the colour, these stimuli thus, possessed the same visual properties, and the behavioral demand was constant across the different conditions (pressing a key). 

### 2.4. EEG Recording and Analysis

The electroencephalograph was recorded while participants performed the task using an Active Two BioSemi system using 64 Ag/AgCL active electrodes were placed on the scalp according to the extended International 10–10 system. In order to record eye movements, electrodes were also placed on the external canthus of each eye. Voltage subtraction across these electrodes was used to screen for horizontal eye movements (HEOG). Signals from an electrode on the inferior orbital region of the left eye was subtracted from the activity recorded at the Fp1 electrode site and provided information about vertical eye movements and eye blinks (VEOG). The EEG and EOG were digitized at 512 Hz and referenced to the average mastoids during analysis. Signals were filtered (low-pass 30 Hz, highpass 0.01 Hz) and were averaged offline. Trials with artifacts at electrodes of interest (Fz, Cz, Pz, PO7, and P08 > 70 *μ*V), eye blinks (VEOG > 70 *μ*V), and large horizontal eye movements (HEOG > 40 *μ*V) were excluded from the analysis. Electrode pooling was applied if needed in order to obtain a clearer signal. EEG was averaged separately for all combinations of conditions. For every trial, EEG epochs of 1000 ms (including a 200 ms prestimulus period) were averaged after artifact rejection. Epochs were then baseline corrected based on mean amplitude of activity recording during the 200 ms immediately prior to stimulus onset. 

To obtain the N1, a time window ranging from 120 ms to 200 ms was used, and the signals from the Oz electrode where used. The P1 and P2 components were also estimated for this electrode site. The time window for the P1 was 60–100 ms and for the P2 was 200–275 ms. 

To obtain the P3a wave, waveforms associated with irrelevant rare trials (no-go same colour) and frequent colour standard trials were averaged separately. Activity for frequent colour standard stimuli was subtracted from the irrelevant rare target averaged signal. The time window that was subsequently used to quantify the P3a mean amplitude was between 280 and 440 ms poststimulus onset. The latency of this component was calculated by taking the most positive point recorded within this predefined time window. The analyses were conducted for the Fz electrode site, which has been shown to present maximal amplitude of this wave on the scalp distribution. By using this same subtraction, we computed the no-go N2. Before averaging, ERPs for every participants were digitally highpass filtered (2 Hz half amplitude cutoff) to remove low frequency generated by the P3s. The time window that was subsequently used to quantify the no-go N2 mean amplitude was between 270 and 293 ms poststimulus onset. The latency of this component was calculated by taking the most positive point recorded within this predefined time window. The analyses were conducted for the Cz electrode site, which has been shown to present maximal amplitude of this wave on the scalp distribution.

We isolated the P3b by subtracting the average signals from frequent position standard trials from the average of rare position trials. The P3b was quantified as the mean amplitude of the subtraction wave between 400 and 750 ms after stimulus. The latency of this component, according to previous study, was calculated by taking the most positive point recorded within this predefined time window. The analyses were conducted on the Pz electrode site, which has been shown to present maximal amplitude of this wave on the scalp distribution. 

To obtain the N2pc and SPCN components, epochs were averaged separately for trials with a right visual field target and trials with a left visual field target. The N2pc component was obtained by subtracting ipsilateral neural activity (recorded over the left hemisphere, PO7, when target stimuli were presented in the left visual field and recorded over the right hemisphere, PO8, when target stimuli were presented in the right visual-field) from contralateral neural activity (recorded over the left hemisphere, PO7, when stimuli were presented in the right visual field and recorded over the right hemisphere, PO8, when stimuli were presented in the left visual-field). For the N2pc, time window ranging between 220 and 270 ms was determined in order to calculate maximal mean amplitude. The SPCN was computed in a time window ranging from 450 to 650 ms. PO7 and PO8 were used to quantify the N2pc and SPCN because this is where these components have the greatest amplitude. 

### 2.5. Statistical Analyses

Latency and amplitude data of each ERP component, as well as behavioral data from the task used for ERP recordings were subjected to descriptive statistics. Student's *t*-tests against zero were also used in order to verify the presence of the evoked components, at a significance level of .05. Also, the topographical distribution on the scalp (topo maps) of each component is presented, in order to demonstrate that the ERP obtained in this study and meet the criteria presented in the literature. 

## 3. Results

### 3.1. Behavioral Results

Behavioral results are shown in Tables 1 to 4. All participants were able to do the task, with a mean accuracy rate of 95% (SD4.24) for the entire task (see Table 1). Reaction times for correct answers are presented in Tables 2 and 3. The mean reaction time was 771.18 ms (SD 163.66) (see Table 4).

### 3.2. Electrophysiological Results

**Figure 2:**
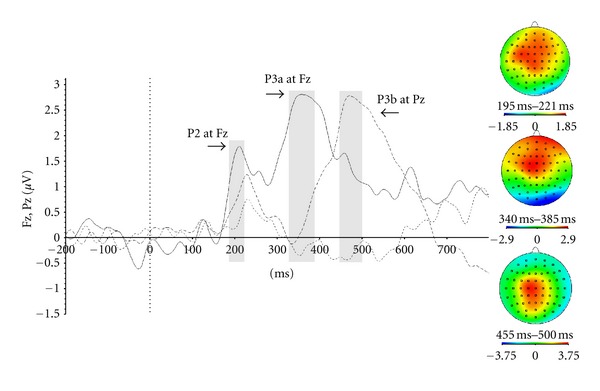
Event-related potentials that isolate the P2, P3a, and P3b componentsfrom averages that do not take into account the side of the target stimulus (in target-present trials). We subtracted the average waveform from the frequent-target, frequent colour, condition (the most frequently occurring condition) from the same-colour (no-go) condition; the solid line shows this difference wave at Fz,while the dashed line shows this difference at Pz. The dotted line shows the difference wave computed by subtracting the frequent-target, frequent colour, condition from the infrequent-target-colourcondition, at Fz which would have been another way to evoke the P3a, according to classic oddball paradigms.

**Figure 3:**
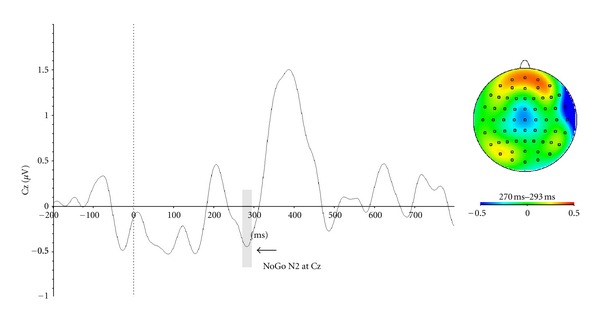
Event-related potentials that isolate no-go N2 component. We subtracted the average waveform from the frequent-target, frequent colour, condition (the most frequently-occurring condition) from the same-colour (no-go) condition; this figure shows this difference wave at Cz.

**Figure 4:**
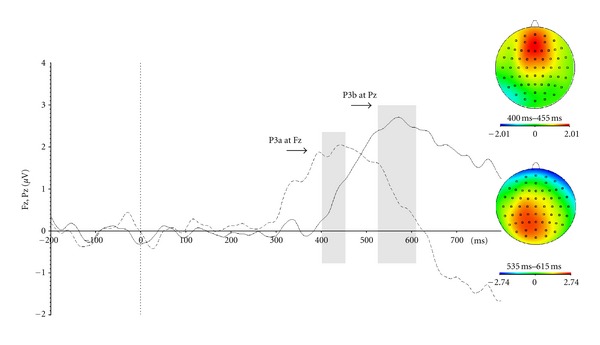
Event-related potentials that isolate the P3a and P3b componentsfrom averages that take into account the side of the target stimulus(in target-present trials). We subtracted the average waveform from thefrequent-target, frequent-position, condition (the most frequently-occurring condition) from the rare-position condition; the solid line shows thisdifference wave at Pz, while the dashed line shows this difference at Fz.

**Figure 5:**
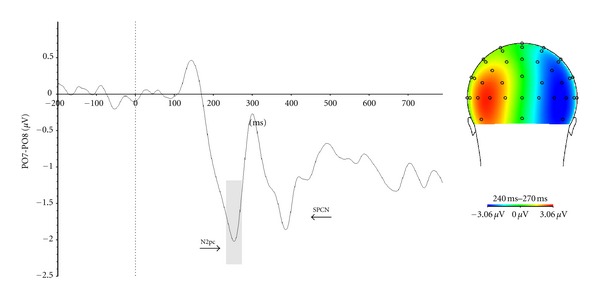
The N2pc component was obtained by subtracting ipsilateral neural activity (recorded over the left hemisphere, PO7, when target stimuli were presented in the left visual field and recorded over the right hemisphere, PO8, when target stimuli were presented in the right visual-field) from Contralateral neural activity (recorded over the left hemisphere, PO7, when stimuli were presented in the right visual field and recorded over the right hemisphere, PO8, when stimuli were presented in the left visual-field). The same subtraction was used to compute the SPCN. To show the scalp distribution of the N2pc, we computed the Grand Average ERP for all trials in which the target was on the left and subtracted from it the Grand Average ERP for all trials in which the target was on the right. As expected if the contralateral response was more negative than the ipsilateral response, the subtraction was positive at left-sided electrodes and negative at right-sided electrodes.

**Figure 6:**
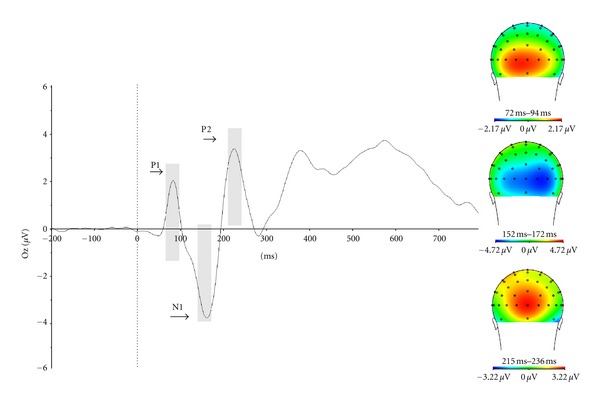
Visual evoked potentials (VEP) obtained by the presentation of infrequent position target. P1, N1, and P2 were maximal at Oz.

*t*-tests against zero were conducted in order to demonstrate the actual presence and reproducibility of the different components. All ERP components were also compared to, and were found comparable to, that expected from the literature in terms of their scalp distribution. The topo maps for each component can be found in Figures 2 to 6. Mean amplitude and significance level for all components of interest are shown in Table 5. 

The first experimental manipulation that aimed to evoke the P3a did not produce the expected results. Indeed, the manipulation in which the participant had to press a key when rare colour target stimuli were presented did not evoke a P3a (see Figure 2 for grand average waveforms). Considering these results, the P3a wave was evoked by coupling the surprise effect created by an irrelevant and rare stimulus, with an inhibition condition created by asking participants not to respond to this type of stimulus. Thus, a completely different type of trial was presented. This different type of trial was composed of the same four squares, all presented in the same colour. These different stimuli appeared in only 10% of the trials, creating a surprise effect. Participants were asked to inhibit their response by not pressing any key when confronted with this type of stimuli. As a result, this condition evoked the P3a.

All components of interest were evoked by this paradigm. They also peaked in the appropriate latency range, as shown in Table 6. 

Figure 3 presents the P3a and its topo map. The mean latency for this component is 440 ms, with maximal activity in frontal sites (Fz, Cz). 

Figure 4 present the P3b component and its topo map. The mean latency for this component is 595 ms, with maximal activity in centroparietal sites (Pz).

Figure 5 present the N2pc and the SPCN complex. The mean latency for the N2pc is 237 ms and for the SPCN is 579 ms. The activity was maximal in the visual areas, more precisely at P07-P08 sites. 

Figure 6 shows the visual evoked potentials at the Oz site. The P1 peaks at 83 ms, the N1 at 151 ms, and the P2 at 234 ms. 

## 4. Discussion

The goal of this study was to create a single task allowing one to follow the various steps of visual information processing from visual analysis to the deployment of visuospatial attention with the use of electrophysiology. Such a task could allow the detection of more subtle functional sequelae related that can remain undetectable on structural neuroimaging, such as those found in mild TBI or mild stroke. This goal has been achieved since we were able to evoke the lower level visual components (P1, N1, and P2), as well as the different visual-cognitive components reflecting the later steps of information processing (P3a, P3b, N2pc, and SPCN). Moreover, those components were evoked using a simple paradigm within a minimal amount of time (about 30 minutes for electrode placement and participant preparation and 30 minutes of testing in the actual task).

This study also brought a new insight into the interpretation of the P3a wave. Indeed, when we attempted to isolate this process by simulating classic oddball paradigm, the P3a was not evoked. Therefore, when the subject has to provide a behavioural response associated with the detection of the rare colour target stimulus, the sole cognitive process consisting in the orientation of attention toward this (irrelevant rare) stimulus appears to be insufficient to elicit a P3a. Other factors thus, seem essential to the generation of the P3a. The no-go trials had two specific characteristics: they were twice as infrequent compared with the rare colour target, and they required response inhibition mechanisms. Although this underlines the importance of the implication of prefrontal and frontal regions in the generation of this component, the exact contribution of withholding the response remains to be determined. Notwithstanding the above, when we added this inhibition condition, we are able to obtain a clear P3a maximal at Fz, peaking between 300 and 500 ms, which corresponds to previous reports in the literature [29].

Over the last few years, many studies were performed in order to develop unbiased and precise indicators of the neurophysiological impairments occurring after a mild brain injury. Some studies conducted on athletes having sustained a mild TBI, using spectroscopy [42, 43], have shown that metabolic impairments were present and measurable many days after a concussion. Other fMRI studies have also shown the link between those impairments and the functioning of the brain [44], especially with respect to the orientation of attention and working memory processes. As shown by these studies, patients with a mild TBI have observable lags in the performance of neuronal networks associated with visual working memory processes. Although such technologies can highlight some of the impacts of mild TBI, their cost make their use relatively unrealistic in dealing with mild brain injuries in a clinical context. 

With its low cost, high temporal resolution, and ease of use, visual and event-related potentials represent an excellent technique to be used for the diagnosis and follow-up assessment of visual and cognitive functions following TBI. Indeed, in particular in mild TBI, it is using electrophysiological methods (visual evoked potentials and event-related potentials) that studies have been able to show alterations in visual information processing, including deficits in complex or integrative visual analysis [8, 45]. Some researchers have even demonstrated a relationship between such deficits and poor functional outcome [8]. However, these studies have generally looked at a single component or process, or have employed separate tasks to assess various steps in processing, making it difficult or more costly to establish the interrelationships between affected mechanisms. The task presented here overcomes this limitation in providing multiple measures which, when analyzed accordingly, permit the evaluation of multiple interdependent mechanisms such as the analysis of visual characteristics (P1, N1, and P2), the deployment and orientation of visual attention (N2pc), visual working memory (SPCN), and cognitive control (no-go N2). Although this last aspect is not significant in this study, future work should try to find an effective way to obtain an objective measure of this cognitive process.

This task does have some limits, however, which are related to its methodological complexity. In order to evoke each of the components of interest, many task conditions had to be included. This led to the rejection of more trials than expected due to artifacts (eye blinks, horizontal saccades, and alpha band) and led to the exclusion from analyses of five participants. It is possible that patients with cerebral impairments, even minor ones, could have some difficulty in performing the task during the acute phase that is, soon after the incident or trauma. On the other hand, the very high accuracy rates in all conditions of the experiment suggest that only severely impaired patients would fail at the task, in which case a sensitive measure of performance is not clinically needed. Halterman et al. [46] have in fact demonstrated that attention orientation and inhibition control impairments were detectable with slower reaction times within from a few hours to a few days after the trauma. Interestingly, this limit brings us back to the clinical reality and to the needs that this task tries to cover. In fact, because the symptoms are generally very important during the few hours following a brain injury, a clinical interview is sufficient to detect cerebral impairment. It is when the physical symptoms fade away, and more subtle sensory and cognitive impairments remain, perhaps unbeknownst to the patient, that there is an important need for more precise diagnostic tools allowing for a good detection of impacted functions and a good followup of recovery. 

 Since studies show that patients can perform as well as controls on the usual clinical tests after the acute phase, we believe that the present multifunction task can be used, with minimal adjustment to reduce artifacts (eg., slightly increase size of stimuli, reduce length of trial blocks and add mandatory interblock pauses), in order to detect lasting and subtle impairments that affect function in day-to-day life. At such later stages, patients will be able to complete the task despite the required attentional effort, and the results could provide information to clinicians on the presence, neurophysiological involvement, and type of impairments reported by the patients. Thus, the next phase of this research work will be to run this task with two clinical populations (mild TBI, mild stroke) and, respectively, matched control groups, in order to establish clinical sensitivity and relevance for detecting the functional visuocognitive impairments that might occur and persist after mild brain injuries.

## Figures and Tables

**Table 1:** Accuracy (percent) for each task condition.

Task condition	Mean	Standard deviation (SD)
Same colour	97.0	3.9
Infrequent position	90.9	5.7
Frequent position	96.4	4.6
Infrequent colour	96.0	3.6
Frequent colour	95.0	5.4
Target in right hemifield	95.4	4.0
Target in left hemifield	94.6	5.1

**Table 2:** Reaction time (ms) for each task condition, for correct answers.

Task condition	Mean	SD
Same colour	No-go trial	No-go trial
Infrequent position	766	129
Frequent position	750	133
Infrequent colour	772	116
Frequent colour	749	137
Target in right hemifield	742	125
Target in left hemifield	765	139

**Table 3:** Reaction time (ms) for each task condition, for incorrect answers.

Task condition	Mean	SD
Same colour	427	381
Infrequent position	800	338
Frequent position	1282	455
Infrequent colour	1152	1097
Frequent colour	1004	365
Target in right hemifield	967	405
Target in left hemifield	1014	467

**Table 4:** Reaction time (ms), for each condition, for correct and incorrect trials.

Task condition	Mean	SD
Same colour	No-go trial	No-go trial
Infrequent position	772	152
Frequent position	771	169
Infrequent colour	780	122
Frequent colour	769	177
Target in right hemifield	760	154
Target in left hemifield	782	175
Task	771	164

**Table 5:** Mean amplitude (*μ*V) for components of interest and their level of significance.

Component	Number of subjects (*N*)	Mean	SD	*t* value	Level of significance (sig.)
P3a	19	2.2	2.4	4.0	.001
P3b	19	1.9	1.5	5.6	.000
N2pc	19	−1.8	0.9	−8.4	.000
SPCN	19	−0.9	1.5	−2.5	.022
N1	19	−2.3	2.7	−3.6	.002
P1	19	1.3	2.4	2.4	.029
P2	19	2.0	3.1	2.8	.011
No-go N2	19	−0.4	1.0	−1.8	.097

**Table 6:** Mean latency (ms) for components of interest.

Component	*N *	Mean	SD
P3a	19	440	94
P3b	19	594	60
N2pc	19	237	28
SPCN	19	579	108
N1	19	151	24
P1	19	83	11
P2	19	234	20
No-go N2	19	273	29
